# Alopecia Areata: Understanding the Pathophysiology and Advancements in Treatment Modalities

**DOI:** 10.7759/cureus.78298

**Published:** 2025-01-31

**Authors:** Yozahandy A Abarca, Renee Scott-Emuakpor, Jhanavi Tirth, Oksana Moroz, George Pandarakalam Thomas, Dana Yateem, Rebecca Golbari, Ninigail Aphia, Yuliya Lysak, Niketa Narasimhan, Humza F Siddiqui

**Affiliations:** 1 Internal Medicine, School of Medicine and Health Sciences, Technological Institute of Monterrey, Monterrey, MEX; 2 Dermatology, University of Miami Miller School of Medicine, Miami, USA; 3 College of Medicine, Smt. Nathiba Hargovandas Lakhmichand (NHL) Municipal Medical College, Ahmedabad, IND; 4 Dermatology, Dr. Andrew Simone - Walk-in Dermatology Clinic, Toronto, CAN; 5 Internal Medicine, Dorset County Hospital, Dorchester, GBR; 6 Dermatology, Shrewsbury and Telford Hospital NHS Trust, Shrewsbury, GBR; 7 Dermatology, Technion American Medical School, Los Angeles, USA; 8 Medicine, Thoothukudi Medical College, Thoothukudi, IND; 9 Medicine, St. George's University, St. George’s, GRD; 10 Biology, Miami University, Oxford, USA; 11 Internal Medicine, Jinnah Postgraduate Medical Centre, Karachi, PAK

**Keywords:** alopecia areata, hair follicles, hair loss, immune privilege loss, immunotherapy, intralesional corticosteroid, jak inhibitor, mesenchymal stem cell therapy, minoxidil

## Abstract

Alopecia areata (AA) is an autoimmune condition that presents with non-scarring hair loss affecting multiple patients worldwide during their lifetime. It ranges from well-defined patchy to diffuse total hair loss, impacting all hair-bearing areas of the body. AA most commonly predominantly manifests on the scalp. The pathophysiology of AA is complex and multi-faceted. The findings of our review article were consistent with the recent literature, delineating autoimmunity, genetic susceptibility, and environmental aspects to be the contributing factors. One of the main causes of AA is believed to be the disruption in the immune privilege of the hair follicles. Multiple genetic loci involved in hair follicle maturation and immune process have been linked to the development of AA as evidenced by several studies. It has been postulated that psychological stressors, smoking, alcohol consumption, sleep disturbances, gut microbiota, and drugs play a role in the pathogenesis of AA by exacerbating the immune response against the hair follicles. AA is a clinically diagnosed disorder. Topical, intra-lesional, and oral corticosteroids, topical and oral minoxidil, cyclosporine, and other immune therapy drugs are widely accepted first-line treatment options, although incomplete remission and relapses are common. Recently JAK-2 inhibitors and mesenchymal stem cell exosomes have shown promising results, potentially treating severe and refractory hair loss. AA has a bidirectional relationship with psychological symptoms as it can lead to social anxiety and depression, which in turn can aggravate hair loss. Hence, it is crucial to implement a holistic approach to managing AA including topical and systemic therapies, psychological counseling, and lifestyle modifications. It is imperative to fully declinate the pathophysiological mechanisms of the disease and formulate therapies in future research to help clinicians and dermatologists devise definitive guidelines to treat the condition for long-term remission.

## Introduction and background

Alopecia areata (AA) is an autoimmune inflammatory disorder that causes a non-scarring type of alopecia, leading to sudden onset patchy or diffuse hair loss [1]. This chronic condition affects hair follicles, nails, and, in some cases, the retinal pigment epithelium [2]. It affects individuals of all ages, genders, and races, with an estimated incidence of 20.9 per 100,000 person-years and a cumulative lifetime incidence of 2% (0.57-3.8%) globally [3]. Children formulate approximately 20% of the cases, while 60% of adults before their 30s report their first patches of hair loss [4]. AA manifests in various forms, which could range from well-defined bald patches to multiple patches, total hair loss of the scalp called alopecia totalis (AT), and a complete loss of body hair called alopecia universalis (AU) [5]. Occasionally, it can present as a band-like hair loss at the periphery of the scalp (ophiasis), involving just the crown (ophiasis inversus) mimicking male-pattern alopecia, sudden greying or AA incognita. Commonly patients experience hair shedding on the scalp, beard, eyebrows, and eyelashes. In addition, 10-40% of patients will experience nail involvement in the course of the disease, which manifests as nail ridging and pitting that correlate with the severity of AA [6].

AA has been associated with autoimmune diseases and other conditions such as asthma, allergic rhinitis, atopic dermatitis, diabetes, hypertension, thyroiditis, systemic lupus erythematosus (SLE), and vitiligo in various studies [7]. Furthermore, AA may be associated with chromosomal disorders such as Down syndrome, and polyglandular autoimmune syndrome type 1 [6]. The exact mechanism that is responsible for the development of AA still needs to be fully elucidated, while the common theory shows the collapse of the immune privilege of the hair follicle caused by immunological mechanisms [1]. The disease is primarily caused when the body attacks its own hair follicles through CD8+ T lymphocytes [8]. Psychological and environmental factors, such as smoking, alcohol consumption, sleep, obesity, fatty acids, and gluten consumption can trigger AA [4]. There is currently no definitive treatment of AA [4]. Treatments for AA consist of various intralesional, topical, or oral corticosteroids, minoxidil, and certain light-based therapies. However, these treatments could come with unfavorable side effects like irritation or unwanted hair growth in other areas of the body. Furthermore, there's no assurance that the newly regrown hair will remain after treatment ends [9]. Novel treatment strategies include Janus kinase (JAK) inhibitors, biologics, several small molecular agents, antihistamines, platelet-rich plasma (PRP) injection, and other therapies [1]. These patients are more likely to suffer from mental health conditions as it can be emotionally devastating leading to low self-esteem, social withdrawal, and depression [10]. Hence, it is imperative to address both the somatic symptoms and psychological aspects during the management of AA [2].

We are conducting this narrative review to gain insights into the clinical presentation, diagnosis, underlying mechanisms, therapeutic approaches, emotional consequences, and emerging trends in research pertaining to autoimmune AA, which help clinicians and dermatologists understand the condition and formulate definitive guidelines for the management of AA.

## Review

Clinical presentation

AA presents with one or a few sudden well-demarcated nonscarring focal hairless patches of round or oval, sharply demarcated shape. The patches are usually symptomless. There is no scale or induration of the scalp and no loss of follicular markings. However, occasionally, patients have some tingling, itching, or dysesthesia, at times preceding the hair loss. Nail abnormalities, such as regular pitting, brittleness, or striations, are observed [11].

The classical AA presents as a single (unifocal) or multiple (multifocal) sharply demarcated patches. It is characterized by a single, round, or oval, with no observable alterations in the skin or hair surrounding the patch. Ophiasic AA presents with hair loss along the line of temporo-occipital implantation, resulting in a widespread hairless area, extending to the inferior margins of the scalp. AT manifests as total loss of terminal hair of the scalp without affecting other body hair. Diffuse AA manifests as acute and widespread hair loss. Most of these cases develop into the more serious AT or AU forms. In AU, there is total loss of body hair, involving the scalp, eyelashes, eyebrows, beard, mustache, and genital areas [12]. Sisaifo type AA (inverse ophiasis) is an atypical manifestation of AA. In this form, the hair loss involves the entire scalp except for the lower margins, along the line of temporo-occipital implantation. It is the inverse clinical image of the ophiasis form. Reticular AA presents with multiple alopecic patches separated by narrow bands of preserved hair, conferring a reticulated aspect to the picture [13].

Yellow dots correspond with distention of the affected follicular infundibulum with keratinous material and sebum and can help demonstrate the persistence of hair follicles, which are typically destroyed in scarring alopecia. Short vellus hairs, which are thin and non-pigmented, correlate with regrowth when seen within AA patches. Exclamation marks hairs are fairly specific for AA. These marks have a wide distal shaft diameter that thins proximally, as a result of the lymphocytic inflammatory infiltrate affecting the hair bulb. Black dots are the result of broken pigmented hair at the level of the scalp. Exclamation mark hairs and black dots are considered signs of active disease [14]. Upright-regrowing hairs and pigtail hairs are positive indicators for hair regrowth, while black dots, broken hairs, exclamation mark hairs, and tapered hairs are considered negative indicators [15].

The Severity of Alopecia Tool (SALT) score involves splitting the scalp into four quadrants and summing the percentage of scalp area devoid of terminal hairs in each quadrant and then the whole scalp to provide a total area affected. It is used to assess the prognosis of the treatment. S0 represents no hair loss, whereas S1, S2, S3, S4, and S5 represent <25%, 25 to 49%, 50 to 74%, 75 to 99%, and 100% hair loss, respectively [16].

Pathophysiology of AA

**Figure 1:**
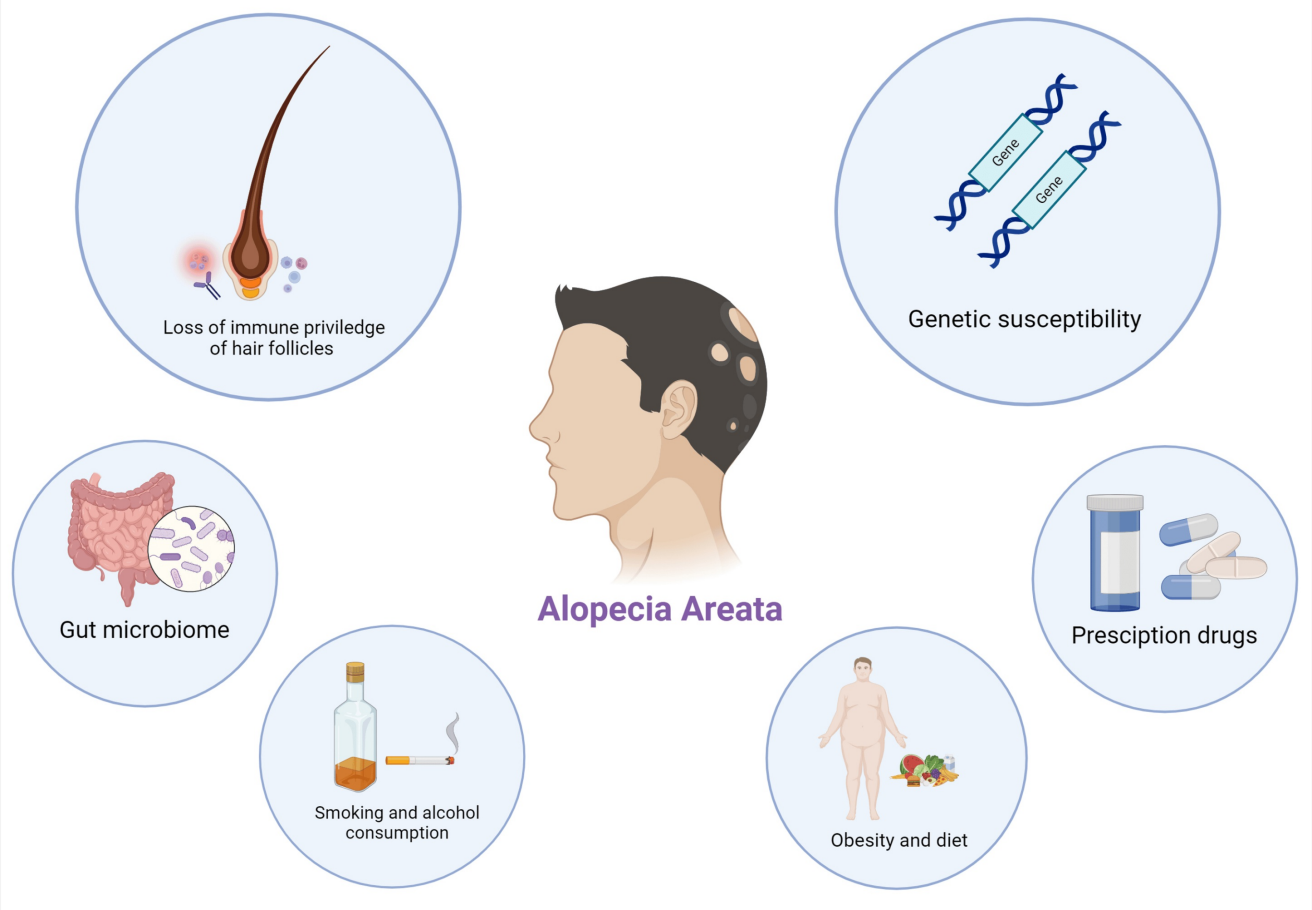
Summary of factors that contribute to pathophysiology of alopecia areata Figure made using biorender.com. Credit: Image created by the authors.

The primary cause of AA is quite intricate [17]. The immune system in humans has a vital involvement in developing AA, through T-cell mediated type IV hypersensitivity reaction. In addition to autoimmunity, genetics and environmental factors including stress and viral infection also have a significant impact on the development of the disease (Figure 1) [18,19].

Immune system involvement

The hair follicles are considered to have privileged immunity, although it is restricted to only the anagen phase 3. The immune system attacks the hair follicles in AA, via two mechanisms. Firstly, by directly attacking the hair follicles, which is generated by the auto antigen by the melanocytes present in the hair follicles, and secondly, by compromising the immune privilege (IP) of the hair follicles due to amplified expression of the major histocompatibility complex (MHC) class I molecule [20].

The activation of CD8+ cytotoxic T cells is initiated by the attachment of a particular auto antigen to MHC molecules on hair follicle cells. Upon activation, they discharge cytotoxic granules, which contain perforin and granzymes. Perforin forms openings in the targeted cell membrane, while granzymes trigger apoptosis in the keratinocytes of the hair follicles, leading to hair loss due to hair follicle destruction. Following this response, certain CD8+ T cells are transformed in the memory T cells, which remain in the body and could trigger a recurrence in AA [2,5]. CD4+ T helper cells, i.e., Th1 and Th17, effector cells secrete cytokines; Th1 produces interferon-gamma (IFN-γ), while Th17 releases interleukin-17 (IL-17) [21,22]. Macrophages are activated by IFN-γ production leading to the boosted immune response. It enhances the expression of MHC through dendritic cells, which eases antigen recognition by T cells, in a similar way IL-17, produced by Th17, boosts and prolongs the inflammatory process. Other ILs such as IL-2 and IL-6 are also elevated in AA. IL-2 promotes T cell proliferation, while IL-6 is responsible for the activation of B cells as well as overall inflammatory response [21,22]. Tumor necrotic factor-alpha (TNF-α) is another key cytokine that advances the recruitment and activation of immune cells, enhances the inflammatory response, and further damages the hair follicles. The interplay between cytokines helps each other to sustain the inflammatory response in AA, e.g., IFN-γ can enhance the effects of TNF-α, leading to increased inflammation. These inflammatory cytokines can disrupt the normal cycle of hair growth, especially the conversion from the anagen to the telogen phase, which causes premature regression and hair loss [19].

The combination of events, i.e., T-cell activation, cytokine release, macrophage activation, etc., leads to a destructive environment for the hair follicles, which results in the weakening of the follicles and hair loss. This inflammatory process disrupts the normal hair growth cycle, which leads to increased shedding and ultimately to patchy baldness. This also results in the destruction of the follicular stem cells, which leads to impaired ability of the hair follicles to recover and regrow hair [19]. In AA, the role of regulatory T-cells is compromised, this may lead to a diminished ability to restrain the activity of effector T-cells, which results in an uncontrolled autoimmune response [18]. On histological examination of the affected tissue, densely infiltrated CD8+ cytotoxic T cells were found, which affects the integrity of hair follicles [20].

Genetic factors

AA is known to have a genetic component to its pathophysiology. Incidence is reported to be higher in those with a family history [23]. Estimate concordance rates of 42-55% have been reported in monozygotic twins [24]. The Genome-Wide Association Studies (GWAS) showed many susceptibilities to loci recognition. Common variation was identified in 14 genetic risk loci [25,26]. HLA-DR is a significant aetiologic driver with the strongest association being MHC [25]. Other HLA genes including HLA-C*04:01 and HLA-DRB1*03 show association with AA [25,27]. Several interleukin genes have also been shown to have significant association with AA [28-30]. The genomic regions associated with AA as shown in GWAS include NKG2D related to natural killer (NK) cell receptors, MICA and ULBP3/6 related to activating ligands, and CTLA4 related to T-cell function regulators [26].

MicroRNAs have diagnostic implications in the lesional expression of AA. It was recently found that miR-101, a microRNA, can act as a diagnostic biomarker for AA [31]. Additionally, there is a significant upregulation of miRNA-155, 146a, and 203 lesional skin expression levels in AA patients. Patients with multiple AA lesions compared to patients with single lesions have significantly higher miRNA-146a lesion expression levels [32]. Key ILs reported as prominent genes by Tishe et al. include IL2RA, IL17A, IL12B, and IL23R genes. The study explores the associations of intronic SNP rs3118470 on IL2RA, rs2275913 on the promotor region of IL17A, rs3212227 in the 3’ untranslated region of IL12B, and rs10889677 in the 3’ untranslated region of IL23R with AA that had been reported in several case-control studies. It showed a significant risk effect for rs3118470 on IL2RA in the dominant and homozygous models with AA and no statistically significant association for the remaining three polymorphisms. The study highlights that the results could be population-specific but require more ethnicity-specific studies to do an ethnic-specific meta-analysis [33].

A whole exome sequencing (WES) and the genome-based collapsing study were done on 849 AA cases and 15,640 controls by Erjavec et al., and KRT82, which encodes hair-specific type II keratin was identified as the only AA case-enriched gene at genome-wide significance. Expression was decreased in the hair follicles and skin of AA patients. KRTCAP3 encoding hair keratin-related protein was identified as a second AA association and DECR2 encoding an enzyme for beta-oxidation was the third most AA-significant gene [34]. Coiled-coil alpha-helical rod protein 1 (CCHCR1) on the AA-associated locus in the HLA class 1 region was identified in a study using next-generation sequencing analysis. It encodes a molecule that constitutes the hair shaft, associating abnormal hair keratinization with AA pathogenesis [35].

A study by Gil-Quiñones et al. did a meta-analysis that showed a significant association between SNP rs2476601 polymorphism of the PTPN22 gene and the risk of developing AA. Further analysis for the homozygous codominant, heterozygous codominant, dominant, and recessive models revealed that the presence of a T allele confers susceptibility to AA. This study, however, found no significant association between rs231775 polymorphism or the CTLA4 gene with AA development. Gil-Quiñones et al. further evaluated that while the roles of rs231775 and rs3087243 variants of CTLA4 are important in the pathophysiology of AA, interpretation should be in accordance with the population due to heterogeneity of results. A meta-analysis was not done for the IL2RA gene due to the heterogeneities of different geographical locations and limited populations included. Meta-analysis for the SNP rs1800682 Fas polymorphism and SNP rs5030772 FASL polymorphism showed no statistically significant association [36].

Environmental factors

Psychological Stress and Immune Modulation

Psychological stress is commonly identified as a trigger for AA; approximately 23% of the patients report an emotional crisis or significant traumatic event preceding the onset of exacerbation of this condition [21]. The skin and brain share a close relationship due to their common origin in the ectoderm. The skin-brain axis facilitates complex interactions between the skin, nervous, endocrine, and immune systems [37,38]. In AA, psychological stress activates the hypothalamic-pituitary-adrenal (HPA) axis, leading to the secretion of neuropeptides such as corticotropin-releasing hormone (CRH) and substance P (SP). These neuropeptides disrupt the IP of hair follicles, which normally protects them from immune system attack [21,39,40]. Environmental stressors and inadequate antioxidant defenses result in an increase in intracellular reactive oxygen species (ROS) within hair follicle cells, enhancing the expression of danger molecules like MHC class I polypeptide-related sequence A (MICA). These molecules are recognized by NK cells, which produce IFN-γ [1,41].

Stress activates both autoimmune and apoptotic mechanisms in the hair follicles. CRH and SP cause keratinocyte death via pathways involving TNF-α, concurrently facilitating mast cell degranulation, which results in neurogenic inflammation [39,40]. This inflammation hastens the transition of hair follicles into the catagen phase, resulting in premature hair loss. SP exacerbates hair growth inhibition by diminishing TrkA receptor activation and enhancing pro-apoptotic pathways, elucidating the connection among stress, telogen effluvium, and AA [40]. A meta-analysis by Toussi et al., which examined 73 studies involving around 414,319 participants, highlights the strong bidirectional relationship between AA and psychological distress. The results indicated a higher incidence of anxiety and depression among AA patients, with stressful life events frequently preceding the onset of the disease. Additionally, more than 80% of AA patients reported a diminished quality of life, particularly in social interactions and self-esteem. Research also indicates that 74% of AA patients will experience at least one mental health disorder in their lifetime​ [42].

Smoking and Alcohol

Studies have shown that smokers have an increased risk of developing AA in comparison to non-smokers. Patients who smoked for more than 10 years or consumed more than five cigarettes per day have a notably higher risk of developing AA. A meta-analysis found a slight but significant association between smoking and AA, with an odds ratio (OR) of 1.12. Free radicals and pro-inflammatory cytokines caused by smoking may contribute to the loss of hair follicles and IP, while pathways involving Th1, Th2, and Th17 responses are central to AA's pathogenesis [43,44]. Alcohol increases pro-inflammatory cytokines, including IL-6, TGF-α, and IFN-γ, which may aggravate illnesses such as psoriasis and potentially AA. It has been postulated that alcohol can increase TH17-mediated inflammation leading to AA. Moderate alcohol consumption can also disrupt stress hormone responses such as adrenocorticotropic hormone (ACTH) and cortisol, influencing AA's pathogenesis [45].

Sleep Disturbances

A cross-sectional study revealed that patients with AA exhibited markedly inferior sleep quality compared to healthy controls, and this diminished sleep quality correlated with elevated levels of anxiety, despair, and reduced quality of life. A separate study emphasized a bidirectional relationship, indicating that individuals with AA possess an elevated chance of experiencing sleep disorders, including obstructive sleep apnea and non-apnea insomnia, and vice versa. Sleep disturbances, objectively assessed by instruments such as the Pittsburgh Sleep Quality Index, were observed to link with elevated stress levels and more severe manifestations of AA. Sleep deprivation adversely affects immunological modulation, resulting in increased inflammation and autoimmune reactions. The precise mechanisms are not well understood; nevertheless, it is probable that disturbances in the HPA axis, which governs stress responses, significantly contribute to this connection [46-48].

Obesity and Dietary Patterns

Obesity contributes to systemic inflammation, potentially via IL-17-mediated pathways, heightening the risk of hair follicle inflammation and the disruption of IP, resulting in hair loss. A study identified a substantial association between elevated BMI and heightened severity of AA. Obesity may exacerbate the progression of the disease and diminish the efficacy of treatments. Lower serum levels of adiponectin, a hormone linked to fat metabolism, were found in patients with AA compared to healthy controls. Additionally, reduced adiponectin levels were associated with more severe manifestations of the disease, suggesting that adiponectin may function as a biomarker of disease severity in obese patients with AA [49,50].

Diets rich in omega-3 fatty acids and antioxidants, such as Mediterranean-style diets, have been effective in reducing systemic inflammation and improving outcomes in inflammatory diseases, including AA. Vitamin D is recognized for its capacity to suppress Th17 cell activity and proliferation, reduce Th1 cell numbers, and enhance T-reg cell function, resulting in immunomodulation. Additionally, vitamin D plays a crucial role as an immunomodulator, and deficiency in vitamin D has been associated with various autoimmune diseases. Retrospective studies comparing AA patients with controls show significantly lower vitamin D levels in AA patients, both in blood and tissue, with an inverse correlation between vitamin D levels and AA severity. A recent study found that vitamin D deficiency is associated with higher C-reactive protein (CRP) levels, and patients with low serum vitamin D and high CRP levels are at a higher risk of developing the universalis form of AA [51-53].

Microbiome Dysbiosis

A study demonstrated that individuals with AA exhibit higher levels of *Cutibacterium acnes *and lower levels of *Staphylococcus epidermidis* and *S. aureus* on the scalp compared to healthy individuals, which may contribute to inflammation and autoimmunity in AA patients. Won et al. recently observed a considerable reduction of *S. caprae* in severe AA, while the *Cutibacterium* species/*S. caprae* ratio increased from 0.97 in healthy controls to 16.01 in severe AA. This ratio could serve as a biomarker for the severity of AA. The involvement of *C. acnes* in AA remains unclear but may be linked to greater disease severity [54-56]. A study by Moreno-Arrones et al. analyzed the gut microbiota of individuals with AA universalis and compared it to healthy controls. The findings indicated that a 25% increase in *Parabacteroides distasonis* and the *Clostridiales vadin BB60 *group was associated with a 9.4% and 11.4% higher probability of developing AU, respectively, suggesting these bacteria could be potential predictors of the condition [57,58].

Viral Infections and Vaccines

The role of viral infections in triggering or exacerbating AA has become particularly evident with the emergence of COVID-19. In their systematic review, Zhu et al. identified cases of AA occurring after COVID-19 vaccination, particularly with mRNA vaccines such as Pfizer and Moderna. The immunological response to the virus and the vaccination can induce the generation of pro-inflammatory cytokines, particularly IFN-γ, which is pivotal in the disruption of hair follicles and IP. The increased secretion of IFN-γ and other inflammatory cytokines following COVID-19 infection or vaccination likely contributes to the exacerbation of AA in susceptible individuals [59]. These findings emphasize the necessity for clinicians to observe individuals with a history of AA for possible illness exacerbations subsequent to viral infections or vaccinations. Moreover, AA has been documented to arise subsequent to infections with viruses like human papillomavirus (HPV), Epstein-Barr virus (EBV), human immunodeficiency virus (HIV), and hepatitis B and C. Certain studies have observed occurrences of AA after hepatitis B immunization [60].

Drugs Causing AA

A cross-sectional analysis using the FDA Adverse Events Reporting System identified monoclonal antibodies as a major drug class implicated in AA, with adalimumab and dupilumab ranking among the top drugs associated with this condition [61]. Among the chemotherapeutic agents implicated in drug-induced alopecia are included docetaxel and capecitabine [62]. A case reported AU following treatment with the angiotensin receptor-neprilysin inhibitor sacubitril/azilsartan used in cardiovascular conditions and thus suggests that therapies aiming at cardiovascular conditions may lead to alopecia as a result of immune modulation [63]. A further case reported AU induced by the B-cell targeting agent rituximab, further supporting the fact that immunomodulatory drugs, especially in more recent times those targeting specific immune cells, can induce alopecia in predisposed individuals [64]. Tyrosine kinase inhibitors, retinoids, phenobarbital, sulfasalazine, rifampicin, and most recently lansoprazole have been implicated in the onset of AA [65,66].

Topical treatments

Topical Corticoid Therapy (TCT)

This treatment involves the usage of creams, ointments, and lotions that contain anti-inflammatory properties that help in follicular regrowth. TCT is said to work effectively for localized bald spots in the initial phase, and the treatment can be further boosted when the medication is secured with a wrap, consequently enhancing the drug absorption in the skin. A study conducted by the Brazilian Society of Dermatology presented the common side effects of TCT, namely folliculitis, skin thinning, and stretch marks. Another very rare but major risk associated with this technique is adrenal suppression because if there is an excess of steroids entering the bloodstream, it can interfere with the hormone levels of the body, leading to potential health complications in the long run [67].

A retrospective, single-centered study conducted in 2024 evaluated the effect of an increased dosage of topical corticosteroids combined with occlusion on younger children with severe AA. Twenty-three children affected by either AU or AT participated in the study. The steroid medications of 0.05% clobetasol propionate or 0.3% diflucortolone valerate are applied to the region of hair loss and then covered with a plastic wrap for a duration of eight hours, six days per week, for six months. The study was primarily measured using a SALT assessment. A SALT score of less than 20 suggested a significant improvement in hair growth. The statistically significant results with a p-value of 0.0001 revealed that 83% of patients achieved a lower SALT score after six months, supporting the hypothesis that a high dosage of TCT with occlusion significantly promotes follicular hair growth. While temporary adrenal suppression occurred in some cases, it was easily reversible, and no other complications were observed throughout the study. The study had various limitations such as a smaller sample size was used, and a lack of a control group and a placebo group suggests an inherent conformational bias [68].

Additionally, another randomized, double-blinded placebo-controlled trial conducted in 2006 evaluated the effect of clobetasol foam (CF) on patients with severe AA. Thirty-two subjects participated in the study, where the CF was applied on the left scalp, and the placebo foam (PF) was applied on the right scalp for five days and 24 weeks. The efficacy of the study was measured using the RGS scale (hair regrowth rate scale). The results revealed that the CF-treated side had a higher growth rate of 89% while the PF-treated side had a growth rate of 11%. This was further supported by a statistically significant p-value of 0.0001, indicating that CF significantly improved hair growth when compared to other ointments and creams. There were major limitations of this study. A smaller sample size was used; topical treatments take a long time to work, but the study had a very short follow-up period, and the longer side effects of CF were not investigated [69]. Research indicates that topical corticosteroid therapy appears to be quite effective in promoting hair growth. The corticosteroid medication needs to be administered precisely, and cortisol levels and ACTH levels need to be monitored continuously for patients using the TCT treatment approach. Lack of monitoring may lead to adrenal suppression, causing potential long-term risk.

As previously mentioned, topical corticosteroids are designed to diminish the onset of inflammation by suppressing the local immune response of the body at the site where the drug is applied. As a result, the inflammation around the hair follicle is reduced, and consequently, hair growth is achieved. However, there are contraindications to the long-term use of corticosteroids. The usage of these drugs for a prolonged period is ill-advised, especially in young children due to the risk of adrenal suppression. Therefore, it is advised that these medications be used with caution, especially for pregnant individuals. Also, skin sensitization or allergic reactions may be possible concerning this medication due to the long-term exposure [67-69].

Topical Minoxidil

Minoxidil is another commonly prescribed topical lotion for hair growth. It can be directly applied to the scalp, and it takes a longer time to show results. Research shows that the topical minoxidil treatment has a minimal effect on reducing AA [70]. A double-blinded placebo-controlled study investigated the impact of different concentrations (1% and 5%) on the hair regrowth rate. Sixty-six subjects were enrolled in the study, with 47 receiving 5% minoxidil and 48 subjects receiving 1% minoxidil. The participants in the experimental group applied the minoxidil lotion twice daily and enhanced the drug absorption using petrolatum. The placebo group was given either of the concentrations for 12 weeks and then shifted to a 5% minoxidil concentration. The RGS scale was used as a primary assessment in this study. The results exhibited statistical significance (p < 0.05) between the treatment groups and placebo groups, revealing that 81% of patients treated with 5% minoxidil observed a significant hair regrowth rate, while 38% of the patients treated with 1% minoxidil observed terminal hair growth. The data from the result were in positive correlation with the hypothesis supporting those higher concentrations of minoxidil significantly improved hair regrowth rate when compared to lower concentrations. The limitations of the study include a smaller sample size and a shorter follow-up period, but the study investigated that minoxidil is a drug that can be easily used with lesser risk and complications [71].

Consequently, another randomized quasi-experimental research study compared the efficiency of intralesional PRP therapy with 5% minoxidil. Forty patients were enrolled in the study and divided into two groups, where one group received PRP therapy and the other group received 5% minoxidil for four months. The SALT score was used to primarily assess the efficacy of the experiment, and the score was monitored from baseline and every month for a duration of four months. The results demonstrated a high statistical difference between the two groups exhibiting intralesional PRP to show better outcomes than minoxidil. Furthermore, the groups treated with PRP therapy had lower SALT scores when compared to the other group, highlighting that intralesional PRP therapy is more efficient in significantly improving hair growth when compared to minoxidil. The major limitations of the study include that the side effects were not systematically recorded, the longer-term effects were not recorded, and the safety protocols were also not investigated. The study used a smaller sample size. All these limitations should also be considered for interpreting the conclusion [72].

An additional double-blinded placebo control investigation reviewed the efficacy and safety of a 3% minoxidil solution for AU and AT. Thirty subjects were randomly assigned to either the treatment group or the placebo group. Subjects applied 3% minoxidil to half of the scalp twice every day, followed by occlusion while sleeping in a yearlong timeframe. The results demonstrated that 63.6% of patients treated with 3% minoxidil experienced improved hair growth, and 35% of the placebo group also experienced hair growth. The statistical significance further supported the data, with a p < 0.05 indicating the higher outcomes of 3% minoxidil than the placebo groups in treating extensive AA. The limitations of the study include only a smaller sample size [73].

Minoxidil does not cause significant side effects but has smaller ones that still occur, such as follicular irritations and mild itching, but long-term risks are unknown. A recurring limitation in all research studies is using smaller sample size and a lack of extended research on the long-term effects of minoxidil. Minoxidil is a vasodilator that increases blood flow to hair follicles, prolonging the anagen phase of the hair cycle and supporting follicular regrowth. Visible results usually need long-term application on a routine basis. It is not recommended to be prescribed in pregnancy and lactation stages or in the case of scalp infections. Furthermore, skin sensitivity may be experienced while using this drug. However, caution is taken in patients with heart conditions because of its vasodilation properties. Common side effects include mild irritation of the scalp accompanied by itching [72,73].

Topical Immunotherapy

Topical immunotherapy involves applying substances like diphenylcyclopropenone (DPCP) or squaric acid dibutyl ester to trigger mild allergic reactions, diverting the immune system from attacking the hair follicles to hair restoration. It can be effective for some patients, while it may cause many allergic reactions like redness, extensive itching, and blisters [74]. A retrospective, single-centered study discussed the effectiveness of DPCP on younger children with severe AA. Initially, a 2% DPCP solution was applied to 4x4 cm, and then varying concentrations of 0.001% to 2% DPCP were applied weekly to induce contact dermatitis. The concentrations were reduced if the patient experienced redness, blistering, or mild itching. The SALT score was used for assessing the hair regrowth rate from the baseline after 6 months and 12 months. The results of the study after six months presented that 3.1% of the patients showed a complete response to the treatment, while 51.5% of patients had no response. There was a slight rise to 8.1% in the response rate after 12 months. The study showed a statistical insignificance of p = 0.002 since the SALT scores were high, subsequently reducing hair growth. The study concluded that immunotherapy has some effect and works only for some people [75].

Another retrospective observational study conducted the same experiment but with a larger sample size to find the efficacy of topical immunotherapy. The results exhibited a success criterion of at least 50% of the patients observing terminal hair regrowth after six months [76]. The recurring limitations in the studies include the failure to measure systematic side effects, leaving gaps in understanding the broader impact of the treatments. This highlights the need for more comprehensive evaluations in future research. Contraindications of this treatment include pregnancy and severe skin conditions such as eczema. Topical immunotherapy should be avoided in patients with any history of severe allergic reactions [75,76]. Topical therapies have been summarized in Table 1.

**Table 1:** Summary of topical treatments

Treatment	Indications	Contraindications	Adverse effects
Topical and intralesional corticosteroid therapy [67-69]	Mild to moderate acute patchy hair loss. First-line treatment for mild to moderate alopecia areata. Suitable for inflammatory skin conditions (e.g., eczema, psoriasis). Used to reduce inflammation and suppress immune response in localized areas.	Active infections on the site of applications. Pregnancy or lactation. Hypersensitivity or allergy to the corticosteroid or any of its components.	Folliculitis, skin thinning, skin sensitization, striae, allergic reactions, hypopigmentation, telangiectasia and hypothalamic-pituitary-adrenal (HPA) axis suppression.
Minoxidil [70-73]	Treatment of androgenetic alopecia in men and women. Maintenance therapy. Combination therapy. Promotes hair regrowth in certain cases of traction alopecia.	Pregnancy and lactation. Scalp dermatitis or skin infections in the treatment area. Children under 18 and adults over 65 without medical supervision. Use on non-scalp areas. Hypersensitivity or allergy to minoxidil or its components.	Hypertrichosis, dryness, postural hypotension, scalp irritation, dizziness, headache, palpitations and swelling in extremities
Topical immunotherapy [74-76]	Chronic case of alopecia. Relapse in the condition.	Pregnancy. Active allergic reaction and other skin conditions.	Redness, severe itching, blisters, flu-like symptoms, lymphadenopathy, hyper or hypopigmentation.

Systemic Treatments

Among systemic treatments for AA baricitinib (Olumiant) is one of the widely available drugs. Its mechanism of action includes inhibition of JAK 1 and 2, which promotes IL-15 cell signaling. Conversely, IL-15 increases the inflammatory response surrounding hair follicles by inducing T cells to produce more IFN-γ. Baricitinib, therefore, seems to be a practical treatment option for AA through blockage of this process. At week 36, a significantly higher proportion of patients achieved a SALT score of ≤ 20 in the baricitinib 4 mg and 2 mg group compared with the placebo group (51.9%, 33.3% versus 3.6%, P = 0.016 and P = 0.001, respectively) that proves high efficacy [77]. The starting dose, 2 mg, daily is advised. If there is not a proper response, then the dose can be increased to 4 mg per day. If the patient has renal impairment, the daily dosage should not go above 2 mg and in fact, should be avoided in this category of patients. Baricitinib is particularly advised for those patients who have not received the desired results through other therapies or if they are suitable for them. In general, the common side effects of baricitinib are infections of the upper respiratory tract, headache, hypertension, and elevation in liver enzymes. Its contraindications include active or serious infections, a history of thrombosis, pregnant patients, and breastfeeding patients. It is also contraindicated in severe liver impairments [77,78].

Another systemic medication that prevents potentially dangerous helper T cell activation in AA is cyclosporine. A pilot study was conducted in which six people with AA (five men and one woman) received oral cyclosporine at a dosage of 6 mg/kg/day for 12 weeks. Three patients had AU, one patient had AT, and two patients had patchy AA of the scalp. All patients noticed an increase in scalp hair throughout the second and fourth weeks of therapy. The growth of hair on the face, chest (in male patients), pubic area, extremities, and axillae was followed. The scalp area was found to have the best response in all patients. In three out of six cases, the terminal hair on the scalp grew back in a way that was aesthetically pleasing. However, every patient had noticeable hair loss [79]. Cyclosporine is usually prescribed as 2.5 to 5 mg/kg per day, depending on the severity, and is divided into two daily doses. Cyclosporine is used in cases of refractory AA because of its side effects profile and can induce significant hair growth especially when combined with other treatments like corticosteroids, etc. Major side effects of cyclosporine include nephrotoxicity, hypertension, hyperlipidemia, and various infections. Contraindications include renal impairment, uncontrolled hypertension, and active infections. However, the use of cyclosporine during pregnancy is contraindicated for possible fetal harm. The drug belongs to category C because animal reproduction studies have shown an adverse effect on the fetus, but there are no adequate and well-controlled studies in humans [80].

Oral minoxidil is used for the treatment of resistant and ongoing forms of AA. Minoxidil is a vasodilator that helps promote and improve the blood circulation of the scalp and, in turn, stimulates hair growth by extending the anagen phase and increasing hair follicle size. In a recent study, it was mentioned that it may lead to a noticeable reduction of 5-alpha reductase type 2 gene expressions. Its dosage can vary between 0.25 and 10 mg per day and the dose can be split one to four times a day based on the type and extent of AA. Adverse effects of minoxidil consist of hypertrichosis, decreased blood pressure, dizziness, fluid retention, headaches, tachycardia, orthostatic hypotension, and swelling of feet and ankles. The dose can range from 0.25 to 10 mg per day and its schedule can differ from one to four times a day based on the severity. It’s more beneficial to use oral minoxidil over topical as its systemic absorption is great that it reaches the hair follicle [81,82].

Mini-pulse glucocorticoid therapy presents an effective safe therapeutic approach for patients with AA refractory compared to topical therapies. Studies show that more than 50% of patients who undergo dexamethasone treatment can achieve a reduction of 50% in SALT score (SALT 50) at nine months [80]. The other study confirms the high efficiency of 16 mg of methylprednisolone orally for two days in a row each week. After three months, 55.6% of patients healed reasonably, and 40% of patients recovered well. 17.8% recovered fairly after six months, while 82.2% recovered well. The recurrence rate was minimal (2.2%), and no adverse consequences were found. For AA, oral mini-pulse methylprednisolone therapy is a quick, safe, and effective treatment option with no negative side effects [83]. Oral dexamethasone is given at a dose of 5 to 10 mg, twice a week on non-consecutive days, while oral prednisolone is administered at 200 mg once in a week or can be split into two doses given twice weekly on non-consecutive days. Mini-pulse glucocorticoid therapy can be utilized for the management of non-responsive AA, as it seeks to alter the immune response. The potential negative effects of glucocorticoid therapy include weight gain, hyperglycemia, osteoporosis, gastrointestinal problems, and a high risk of infection. Extended use of glucocorticoids may also result in mood swings, cataracts, and glaucoma. Glucocorticoids are not advised for patients with a history of peptic ulcer history, uncontrolled diabetes, severe cardiovascular disease, and those who are at risk of infections or have undergone recent surgery due to aggravation of symptoms. As stated earlier, glucocorticoids function as immunosuppressants, hence, it is unsuitable for those with active tuberculosis and herpes simplex. In addition, patients with a previous history of psychiatric disorders must be carefully monitored during therapy due to the mood changes that the medication can induce [84].

Mesenchymal stem cell exosomes have been studied to promote hair development as an alternative systemic treatment for AA. It is administered as a drug in the form of a detachable microneedle patch. Mainly made of hair keratin has the ability to combine with exosomes of human bone marrow mesenchymal stem (BMSC) and a small molecular drug called UK5099 to cause pigmentation and hair growth in mice [45]. The most recent exosome study is a retrospective analysis in South Korea, which indicates that 12 weeks of adipose-derived stem cell-derived exosomes (ADSC-exos) treatment resulted in considerable improvements in hair density and thickness in 39 alopecia patients [85]. It is administered as a drug in the form of a detachable microneedle patch. One to three sessions spaced four to six weeks apart are suggested but they depend on the severity of the case. The complications arising from stem cell treatments can include local reactions at the site of injection, inflammation, and probable immune system activation. Relative contraindications include patients with autoimmune diseases, active infections, and allergies to any component used in the treatment [86,87]. Systemic therapies have been summarized in Table 2.

**Table 2:** Summary of systemic treatments JAK: Janus kinase; eGFR: estimated glomerular filtration rate; OAT: organic anion transporter; DMARDS: disease-modifying anti-rheumatic drug; DVT: deep venous thrombosis; LDL: low-density lipoprotein; HDL: high-density lipoprotein; BCG: Bacillus Calmette-Guérin

Treatment	Indications	Contraindications	Adverse effects
Oral corticosteroids [83,84]	Rapidly progressive alopecia areata	Active fungal or viral infections. Severe diabetes. Uncontrolled hypertension. Severe osteoporosis. Peptic ulcer disease. History of steroid-induced psychosis. Heart failure. Cataracts and glaucoma.	Weight gain, hypertension, swelling, fatigue, muscle weakness, thinning of skin and striae and fractures.
JAK inhibitors (baricitinib) [77,78]	Severe alopecia areata with more than 50% hair loss	Severe active local or systemic infections. Chronic kidney disease with an eGFR <30 mL/min/1.73 m². Anemia with hemoglobin levels below 8 mg/dL, lymphopenia with an absolute lymphocyte count <500 cells/mm³. Pregnancy. Administration concurrently with OAT3 inhibitors and other DMARDS or JAK3 inhibitors.	Bone marrow suppression leading to anemia, neutropenia, and lymphopenia, elevated risk of severe infections, increased risk of lymphoma and other malignancies, DVT, pulmonary embolism, and arterial thrombosis, increased susceptibility to herpes zoster infections and increase in mean cholesterol, LDL, HDL.
Cyclosporine [79,80]	Severe alopecia areata	BCG attenuated vaccine hypersensitivity to cyclosporine or any of the ingredients of the formulation. Active infection. Patients with impaired renal function. Asthma. History of blood dyscrasias. Uncontrolled hypertension.	Hypertension, arrhythmia. Decreased GFR, dyslipidemia, convulsions, encephalopathy, anxiety, headache, and fever. Increased occurrence of malignancies and infection.
Mesenchymal stem cell (MSC) exosomes [85-87]	Severe and refractory alopecia areata	Active Infection: Active cancers or in patients with a history of malignancy. Pregnancy. Autoimmune disorders.	Immunological reactions including mild fever, chills, or allergic reactions following exosome administration. Thrombosis, headache, nausea, and fatigue.

Psychological impact and counseling

AA may cause anxiety, depression, isolation, distress, and paranoia. AA often has psychosocial effects that may impose an emotional burden on the affected individual. Hair loss on the scalp, eyebrows, and body can deeply impact a person's perception of their body image, identity, and self-worth. Because hair loss is visible, it leads to social withdrawal and alienation. Hair loss can cause significant emotional distress and serves as a persistent reminder of the patient's diagnosis. This heightened awareness often increases feelings of anxiety and depression, which makes it difficult for individuals to participate in social and work settings due to insecurities and discomfort. Therefore, the disease's physical symptoms may lead to psychological scarring, and this may cause further exacerbation. Therefore, mental counseling is grossly needed when treating individuals diagnosed with AA. AA affects nearly 2% of the global population and occurs in all age demographics and ethnic groups. Although children and young adults must confront the noticeable changes in appearance head-on, the manifestation of the disease can affect anyone. However, early treatment and therapy can reduce the disease's physical and emotional harm. A meta-analysis suggested that 13% of individuals diagnosed with AA suffer from some form of anxiety, which is once again higher than the general population. Furthermore, about 34% of AA patients display a physical form of anxiety-driven behaviors. This anxiety could stem from a fear of societal judgment or relapses, thus incurring a vicious cycle [2]. Experiencing anxiety may cause more hair loss and alopecia replated flare-ups. Anxiety surrounding AA regarding children may stem from social consequences [88,89].

Children often get victimized, which challenges their self-esteem and causes lasting trauma. Psychological treatments such as cognitive behavioral therapy (CBT) and mindfulness-based stress reduction are effective in reducing anxiety in AA patients. Furthermore, early implementation of support-rich psychological approaches and various treatments may benefit patients diagnosed with AA. Comprehensive treatment must focus on psychological support as well as holistic interventions. The findings further suggest that support groups and counseling may benefit patients diagnosed with AA. These interventions may further assist patients in implementing unique coping strategies and adjustment tools for managing the emotional upheavals incurred with a diagnosis. Living with a condition that sets one apart from their peer group can be difficult and challenging. It is especially evident in those who began suffering from it at an early age. Adolescent AA patients are most prone to social issues. The cultural stigma around hair loss could worsen this lack of confidence. In some societies, people equate hair with beauty and identity. These social challenges increase the risk of poor mental health, including depression. Societal pressures may make individuals feel worthless and harm their self-esteem. This struggle for acceptance leads to self-criticism, leaving individuals feeling isolated, frustrated, and helpless because of the condition. Patients with a long history of hair loss might say it impacts their daily lives, job prospects, and relationships [89,90].

General paranoia may add to the burden of AA. Diagnosed individuals often feel judged by their appearance, therefore, their fear of judgment reinforces isolation. It prevents patients from seeking support or discussing their problems. This, in turn, adds to their distress. Paranoia is a state of mind. It will arise in patients who think others are observing their alopecia. This will make them hypersensitive to social cues that may not exist and can worsen depression and anxiety. Hypervigilance and paranoia harm supportive social ties. CBT and mindfulness-based methods can reduce paranoia. They help people reframe negative thoughts and manage emotions in social situations. The goal of psychological management is to improve self-esteem, reduce social isolation, and address emotional and cognitive issues. This can relieve depressive symptoms and significantly enhance the quality of life for people with AA [89,91].

Future perspectives

Future research in the treatment of alopecia can highlight various areas to understand both the mechanism of alopecia and treatment options. Research can further explore JAK inhibitors. While JAK inhibitors have shown great success in helping hair growth in patients suffering from alopecia, there are still many unknowns. Much of the current research involves short-term studies. Therefore, long-term studies are necessary to understand possible side effects and the ideal duration of therapy. Furthermore, it would be beneficial to detect biomarkers that may predict which patients are likely to profit from JAK inhibitors. This could help personalize therapy for patients and lead to higher rates of success with therapy as well as avoiding unnecessarily treating patients with medication with potential adverse effects. Additionally, the development of topical formulations of JAK inhibitors would be beneficial in promoting hair growth while also minimizing adverse side effects. Additional research into stem cell therapy is a thrilling possibility. Previous research has noted that hair follicles contain stem cells; however, further studies are needed to better understand the signaling pathways that lead to their initiation and differentiation. Future research can explore current knowledge on molecular signals such as the bone morphogenetic proteins (BMP), Wnt, and Hedgehog pathways. Since it is currently understood that alopecia is an autoimmune condition, future research can further explore the immune response to identify specific immune targets in therapy. One possible objective in potential research can focus on promoting immune tolerance to hair follicles. By encouraging hair follicles to not recognize hair follicles as foreign, it could prevent the autoimmune attack thus preventing further hair loss. A prospective path in research could focus on improving nanotechnology to improve the delivery of drugs. Nanotechnology can directly deliver drugs straight to hair follicles. This can heighten efficacy while reducing unfavorable side effects. The improvement of nanotechnology can enhance the effectiveness of current treatments already in use such as JAK inhibitors and minoxidil. This can also be useful by making current treatments safer for long-term use. There are many promising avenues that future research can focus on to prevent hair loss and regenerate hair follicles to improve the quality of life for patients suffering from alopecia [92-94].

## Conclusions

AA is a clinically diagnosed condition with partial to complete hair loss. It has a multifactorial etiology with autoimmunity, genetic susceptibility, and environmental factors playing a significant role in the development of the disease. Corticosteroids remain the first line of therapy, although there is a lack of complete remission and a risk of relapse. Emerging treatment modalities have shown promising results. However, it is important to devise individualized strategies to ensure patient satisfaction and enhance quality of life. There is an emergent need to conduct further research to completely understand the pathophysiology and develop novel management therapies for long-term remission.
